# Cross-Species and Human Inter-Tissue Network Analysis of Genes Implicated in Longevity and Aging Reveal Strong Support for Nutrient Sensing

**DOI:** 10.3389/fgene.2021.719713

**Published:** 2021-08-27

**Authors:** Avijit Podder, Anish Raju, Nicholas J. Schork

**Affiliations:** ^1^Department of Quantitative Medicine, The Translational Genomics Research Institute (TGen), Phoenix, AZ, United States; ^2^Department of Population Sciences and Molecular and Cell Biology, The City of Hope National Medical Center, Duarte, CA, United States

**Keywords:** human aging, cross-species orthologs, protein-protein interaction network, pathway enrichments, nutrient sensing, drug targets

## Abstract

Intensive research efforts have been undertaken to slow human aging and therefore potentially delay the onset of age-related diseases. These efforts have generated an enormous amount of high-throughput data covering different levels in the physiologic hierarchy, e.g., genetic, epigenetic, transcriptomic, proteomic, and metabolomic, etc. We gathered 15 independent sources of information about genes potentially involved in human longevity and lifespan (*N* = 5836) and subjected them to various integrated analyses. Many of these genes were initially identified in non-human species, and we investigated their orthologs in three non-human species [i.e., mice (*N* = 967), fruit fly (*N* = 449), and worm (*N* = 411)] for further analysis. We characterized experimentally determined protein-protein interaction networks (PPIN) involving each species’ genes from 9 known protein databases and studied the enriched biological pathways among the individually constructed PPINs. We observed three important signaling pathways: FoxO signaling, mTOR signaling, and autophagy to be common and highly enriched in all four species (*p*-value ≤ 0.001). Our study implies that the interaction of proteins involved in the mechanistic target of rapamycin (mTOR) signaling pathway is somewhat limited to each species or that a “rewiring” of specific networks has taken place over time. To corroborate our findings, we repeated our analysis in 43 different human tissues. We investigated conserved modules in various tissue-specific PPINs of the longevity-associated genes based upon their protein expression. This analysis also revealed mTOR signaling as shared biological processes across four different human tissue-specific PPINs for liver, heart, skeletal muscle, and adipose tissue. Further, we explored our results’ translational potential by assessing the protein interactions with all the reported drugs and compounds that have been experimentally verified to promote longevity in the three-comparator species. We observed that the target proteins of the FDA-approved drug rapamycin (a known inhibitor of mTOR) were conserved across all four species. Drugs like melatonin and metformin exhibited shared targets with rapamycin in the human PPIN. The detailed information about the curated gene list, cross-species orthologs, PPIN, and pathways was assembled in an interactive data visualization portal using RStudio’s Shiny framework (https://agingnetwork.shinyapps.io/frontiers/).

## Introduction

The aging process is affected by a number of interconnected genetic, epigenetic, metabolic, and environmental factors; however, their exact degree of contribution is unclear as there is no universally accepted theory of aging (Salnikov and Baramiya, 2020) within which their contribution can be put into context. Several molecular mechanisms such as telomere shortening and cellular senescence, defective autophagy, and DNA damage have been shown to play a critical role in driving aging and related diseases (López-Otín et al., 2013). Selected treatments like the repurposed drug metformin have been demonstrated to effectively slow down or reduce some hallmarks of aging (Haynes, 2020; Kulkarni et al., 2020; Melzer et al., 2020). Therefore, a thorough understanding of aging-related mechanisms is essential to design effective interventions and reliable longevity and healthy-aging promoting strategies. In this regard, the inspection of evolutionarily conserved aging genes in different species and the anti-aging interventions they may motivate, such as caloric restriction, can reveal functionally important cross-species gene modules worthy of further study (Ma and Gladyshev, 2017; Komljenovic et al., 2019). Some efforts to enable this have been undertaken previously, but they have succeeded only partly because an integrative analysis of several underlying biological pathways and metabolic processes was challenging to pin down potential therapeutic targets (Kirkwood, 2011).

The advent of high-throughput technologies has led to the generation of enormous amounts of large-scale data from various cellular levels ranging from genomics to proteomics across different tissues and species. The availability of such technologies provides an excellent opportunity for studying aging through an integrative systems biology approach (Beltrao et al., 2010; Després, 2020; Melzer et al., 2020). In this light, genes and proteins do not function in isolation; instead, they function as part of a complex network of interacting (physical or indirect) components, which, if perturbed, often lead to detrimental physiological changes (Vidal et al., 2011; Sahni et al., 2013). Evaluating the influence of specific perturbations on this network of biological interactions can reveal underlying vulnerabilities in the aging process (Beltrao et al., 2010; Safari-Alighiarloo et al., 2014; Liu et al., 2020). For instance, network-based analyses have been instrumental in identifying proteins that serve as “hubs” that influence numerous other proteins residing within or outside a particular pathway. They can also locate novel “disease modules” composed of previously unknown genes and factors that determine or substantially control a disease state (Chan and Loscalzo, 2012; Marín et al., 2019). In addition, advanced methods exploring disease-dependent expression profiles of the studied proteins with their corresponding drug-dependent profiles have led to insights into effective drug targets (Lamb et al., 2006; Subramanian et al., 2017; García del Valle et al., 2019).

In the present work, we studied genes that have been reported to influence human aging and longevity. We used a network approach to decipher these genes interactions with physical protein-protein interaction networks and further investigate their properties in three different, evolutionary distant, species (i.e., mice, fruit fly, and *C. elegans*). We found three critical signaling pathways: FoxO signaling, mTOR signaling, and autophagy, to be highly enriched (adjusted *p*-value ≤ 0.001) and shared between humans and all three other species. We found evidence for rewiring of specific protein networks of the mTOR signaling pathway across the species we studied. We also exploring whether or not our findings could shed light on human tissue-specific PPINs by correlating the tested genes’ mRNA and protein levels across 43 different human tissues using publicly available data. We found that highly expressed proteins were less likely to be the hub proteins in human tissue-specific networks. To explore our findings’ translational potential, we retrieved all experimentally verified drugs and compounds reported for longevity in the non-human species we studied and evaluated the potential targets of the aforesaid drugs for each species, including humans, using available drug-protein interaction information by determining the positions of these targets within their respective species-specific PPINs. Together, our results could benefit future studies considering effective combination therapies for age-related diseases, drug repurposing, or the development of novel therapeutics for enhancing longevity in humans.

## Materials and Methods

### Data Curation and Filtration

We manually curated a large list of genes hypothesized to be implicated in human longevity and aging from 15 publicly available databases (Table 1). The databases were initially categorized based on the following criteria: SNP association (GWAS Catalog, dbGAP and GAD); Disease or phenotype information (GHMD, HPO, DisGeNet and Open Targets); pathways and functional information (KEGG, AMIGO); curated list of aging and longevity genes (Morbid Map, GeneAge and LongevityMap); literature-based information using PubMed and MEDLINE search (NCBI-MeSH Unique ID: D000375 and D008136); and DNA methylation and gene expression based signatures of human aging-associated genes from the “Aging clusters” resource and the Digital Aging Atlas Database. The genes associated with the keyword “human longevity” from GWAS Catalog, dbGAP, GAD, and OMIM Morbid Map databases were curated through the Harmonizome database (Rouillard et al., 2016). The “Aging clusters” resource is a large-scale data set with meta-data on 6600 human genes combined from 35 datasets that cover aging hallmarks, longevity, changes in DNA methylation, gene expression, and different age-related diseases (Blankenburg et al., 2018). The Digital Aging Atlas (DAA) is a collection of 2599 human age-related genes examined in different biological settings such as molecular, cellular, physiological, psychological and pathological settings, meant to enhance, and expand genetic insights into human aging (Craig et al., 2015).

**TABLE 1:** Databases used for aging and longevity associated genes curation.

Databases	Type of data	No. of genes	Source
GWAS catalog	SNP genotype	230	https://www.ebi.ac.uk/gwas/
dbGAP	SNP genotype	32	https://www.ncbi.nlm.nih.gov/gap/
GAD	SNP genotype	102	https://geneticassociationdb.nih.gov/
GHMD	Disease/phenotype	33	http://www.hgmd.cf.ac.uk/ac/index.php
HPO	Disease/phenotype	283	https://hpo.jax.org/app/
DisGeNet	Disease/phenotype	89	https://www.disgenet.org/
Open Targets	Disease/phenotype	306	https://www.targetvalidation.org/
KEGG	Functional	385	https://www.genome.jp/kegg/
AMIGO	Functional	310	http://amigo.geneontology.org/amigo
Morbid Map	Curated	358	https://omim.org/
GeneAge	Curated	1780	http://genomics.senescence.info/genes/
LongevityMap	Curated	863	https://genomics.senescence.info/longevity/
NCBI-Mesh	Literature driven (PubMed and MEDLINE)	536	https://www.ncbi.nlm.nih.gov/mesh/
DNA Methylation	Aging clusters + Digital aging Atlas	200	http://aging-map.org/, https://gemex.eurac.edu/bioinf/age/
Gene Expression	Aging cluster + Digital aging atlas	308	http://aging-map.org/, https://gemex.eurac.edu/bioinf/age/

*A total of 15 publicly available independent databases were used together to identify human aging and longevity related genes. Genes present at least in two databases (*N* = 1007) are selected for the analysis.*

The total number of genes included from each database are listed in the Table 1. We initially identified 5836 genes from these sources, but after removal of duplicate gene names a total of 4060 non-redundant genes were considered in our analyses. Genes that were listed in at least 2 of these sources were selected (*N* = 1007) for further analyses (Supplementary Table 1). Further, we implemented a Conserved Evidence Scoring (CES) method describe by Kanduri (2019) to rank the genes in our study:

$$\text{GeneRank}:\text{CES}\,\left(G\right)={\text{G}}_{\mathrm{L1}}+{\text{G}}_{\mathrm{L2}}+\cdots +{\text{G}}_{\mathrm{Ln}}/\text{n}\,\left(L\right)$$

Where the CES of a gene (G) was calculated based on the available evidence that a gene is associated with longevity or aging across the 15 available datasets (L_1_, _2 … 15_) used in our study (Supplementary Table 1). We recognize that information in some data sets, although curated at some level, may not be as reliable as others and that longevity and aging are obviously related but not synonymous. However, we purposely chose to cast a wide net in the genes we considered in our studies.

### Mapping Orthologous Genes Across Species

The orthologs of 1007 human genes associated with aging and longevity were mapped against three comparator species (e.g., mice, flies, and worms) using “gene to gene” (i.e., 1:1) ortholog mapping from the Ensembl database using the g:Profiler web tool (Raudvere et al., 2019). We also obtained the ortholog information of 1007 genes across 56 other non-human species using the “gene to OGs (orthologous groups)” mapping tool from the OrthoDB database (Kriventseva et al., 2019). A Conserved Orthologs Score (COS) was further calculated for each gene using the method described by Kanduri (2019) and considered for all the 56 other non-human species in additional analysis (Supplementary Table 2).

### Physical Protein–Protein Interaction Networks (PPIN)

The raw physical protein-protein interactions (PPIs) information for human and mice, flies, worms were obtained from the results of experiments reported in 9 curated databases (BioGRID, IntAct, I2D, MINT, InnateDB, DIP, HPRD, BIND, and BCI) and processed with the Integrated Interactions Database (IID) (M Kotlyar et al., 2019). We characterized experimentally determined protein-protein interaction networks (PPINs) involving the genes for each species using an open-source software platform Cytoscape v3.7.1 (Shannon et al., 2003). To understand the topology of the overall networks, we computed metrics such as node degree (or connectivity), betweenness centrality (BC), and closeness centrality (CC). All these metrics were calculated with NetworkAnalyzer (Assenov et al., 2008) which is a Java plugin for Cytoscape.

In addition, we also constructed a broader physical PPIN, also term as “local-PPIN” for the 1007 aging and longevity associated genes in humans from three available human protein interactome resources: IID (Kotlyar et al., 2019), MIST (Hu et al., 2018), and HuRI (Luck et al., 2020). A union of all three interactomes further identified 911 genes that are likely to exhibit physical interactions with other proteins (Supplementary Table 3).

### Tissue-Specific Transcripts and Protein Expression Profiling

The mRNA (transcript) abundance of the 1007 genes were curated using two public domain databases: the Genotype-Tissue Expression (GTEx) (GTEx Consortium, 2013) database and the Human Protein Atlas (HPA) (Uhlén et al., 2015). We obtained **t**he normalized transcripts per million (NX-TPM) values for 987 and 1001 genes in 34 and 43 different human tissues, respectively, available from each database. The 1007 genes were ultimately evaluated for four different tissues (adipose, heart, liver, and skeletal muscle) based on the primary cell type information in the HPA databases, with variations in protein expression level of the genes assigned as high, moderate, weak and negative as described in the available annotations within the HPA database.

### Functional Enrichments Analysis

A gene-set-based functional enrichment analysis was performed for human and mice, flies and worms using the g:GOSt tool from g:Profiler website^1^. We used gene and pathway annotations in two common data sources for this: the Gene Ontology (GO) and Kyoto Encyclopedia of Genes and Genomes (KEGG) databases. Actual enrichment statistics and *p*-values were calculated by applying a two-sided hypergeometric test followed by a Bonferroni correction to the resulting *p*-values (*p* < 0.05) to identify enrich or overrepresented pathway and terms in the respective databases (Reimand et al., 2019). Relevant gene ratio statistics were calculated by subtracting the intersection size with the term size (i.e., the number of genes representing a particular pathway or other term in GO and KEGG databases).

### Longevity Drugs and Targets Across Species

We created a list of reported drugs and compounds which have been experimentally verified to promote longevity in mice, fruit fly and *C. elegans* from DrugAge database (Barardo et al., 2017). We identified the potential targets of the aforesaid drugs for each species, including humans, using available drug-protein interaction information from STITCH v5.0 database (Szklarczyk et al., 2016). Experimental protein–chemical interactions with confidence score cut-off >0.90 were used to identify the putative targets from STITCH database.

### Cross-Validation of the Curated Gene-List

We performed a PubMed literature-based information search and extraction for genes associated with human longevity and aging using the medical subject heading (MeSH) Unique ID (D008136 and D000375) from BioLitMine database (Hu et al., 2020). Further, all the genes in the curated list (including the 1007 studied genes) were mapped against the available PubMed literature to verify their links with human longevity and aging.

### Data Visualization and Availability

The PPINs we generated were visualized and considered in further analysis using the Cytoscape v3.7.1 software platforms (Shannon et al., 2003). Analysis was conducted in R (R Core Team, 2014) and figures reflecting the results of our PPIN constructions were produced using the package ggplot2 (Wickham, 2009). Additional details about our curated gene list, cross-species orthologs, PPIN, and pathway constructions are available using the link^2^ : which includes interactive visualizations created using RStudio’s Shiny framework (Chang et al., 2021).

## Results and Discussion

### Integrated Resource for Genes Related to Human Longevity and Aging

We first sought to improve curation of lists of longevity and aging-related genes knowing that longevity and aging are complex, multifactorial processes which defined by precise interactions and the molecular integrity of the genome, epigenome and proteome over the time (Carmona and Michan, 2016) which might be hard to characterize. Although significant effort has been made in the past to generate databases and resources capturing genes identified as longevity and aging-related from reviewing the primary literature for human and non-human species (Tacutu et al., 2010, 2018), there is still a lack of consensus of what criteria and curation procedures should be used for generating lists of longevity and aging-related genes. In addition, some resources have resulted from a more comprehensive and systemic integration of longevity and aging-related data at various levels (Craig et al., 2015; Blankenburg et al., 2018). However, such resources have limited agreement with other resources in terms of overlapping or common genes.

Our approach involved extracting and integrating information from 15 different resources. We identified 4060 non-redundant genes from publicly available databases focusing on genotype and longevity and aging phenotype associations, functional context with respect to longevity and aging, DNA methylation patterns associated with longevity and aging and gene expression signatures derived from studies contrasting long-lived and short-lived individuals as well as older and younger individuals. We computed a conserved evidence score (CES) to rank each gene based on its evidence for being implicated in longevity and aging across the 15 resources for 1007 genes that were listed in at least two of those resources. These genes, listed in Supplementary Table 1, were used in subsequent analyses).

### Identification of Orthologs for Longevity and Aging-Related Genes in Non-human Species

Aging affects different species in different ways and this fact contributes to the variation in lifespan exhibited by different species. However, many studies have shown that modulating genes in non-human species can affect the lifespan of those species and therefore provide a rationale for studying the human orthologs of those genes in studies of human longevity and aging (Partridge, 2011; Muntané et al., 2018; Singh et al., 2019). Thus, it is important to understand the evolutionary history of a gene, often measured by its sequence and functional conservation across species, to determine if gross changes to that gene, or its regulatory elements, may have occurred over evolution that have possibly changed its effect on longevity and aging in a different species. Genes that appear to be more highly conserved across species, and therefore have more obvious orthologs in other species, tend to have very fundamental and important biological functions likely to impact longevity, aging, and the overall susceptibility to disease of species (Georgi et al., 2013). Thus, identifying, e.g., highly conserved human orthologs of those genes exhibiting strong associations with longevity and aging in a non-human species provide candidate genes for study in human studies of longevity and aging (Murabito et al., 2012; Zhang et al., 2016).

**FIGURE 1:**
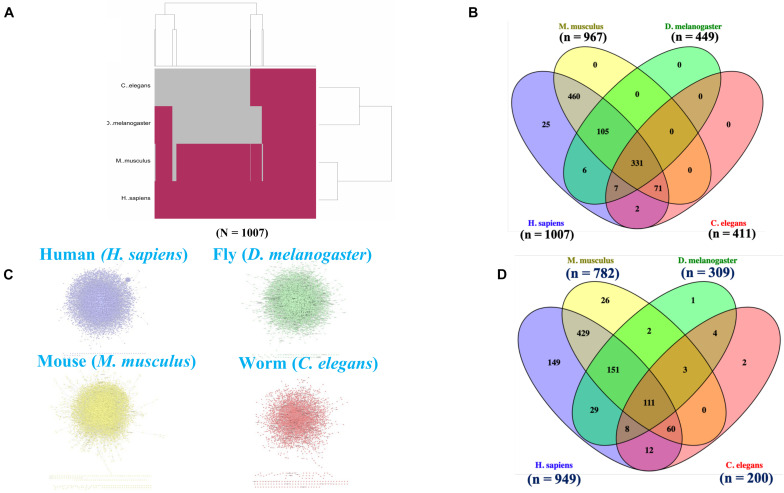
Cross-species orthologs and protein-protein interaction network (PPIN) mapping. **(A)** A hierarchical clustering highlighted the orthologous for the 1007 human aging and longevity related genes in three model organisms (e.g., mice, flies, and worms) by “Gene to Gene” (1:1) orthologs mapping using Ensembl Gene ID. **(B)** Venn diagram shows an overlapping of 331 orthologs genes within the four species. The (n) indicates the number of orthologous genes present in each respective species. **(C)** The protein interactomes for human, mice, flies and worms are curated using Integrated Interactions Database (IID) based on experimentally detected Protein-Protein Interactions (PPIs) from 9 curated databases (BioGRID, IntAct, I2D, MINT, InnateDB, DIP, HPRD, BIND, and BCI). **(D)** Venn diagram shows an overlapping of 111 orthologs genes within the PPIN of four species. The (n) indicates the number of orthologous genes present in respective PPIN of each species.

We identified the human, mouse, fly, and worm orthologs of the 1007 human genes that we considered as associated with longevity and aging. Many of these genes were initially identified as associated with longevity and aging in non-human species and ultimately yielded 967 (of the 1007) human gene orthologs of mouse genes, 449 human orthologs of fruit fly genes, and 411 human orthologs of *C. elegans* genes (see Figures 1A,B). 331 genes were identified as common orthologs across all four of the species studied, and many of the 1007 genes exhibited orthologs in more than two of the four species. We note that the total number of orthologs involving humans are greatly reduced when higher (human and mice) are compared to lower (fruit fly and *C. elegans*) organisms, which is expected likely a result of gene loss events, duplication (paralogous genes) events, and evolutionary constraints acting on the species (Georgi et al., 2013).

To obtain a much broader picture about evolutionary relationships of the 1007 genes, we obtained information about orthologous genes from the OrthoDB database for 57 different species (Kriventseva et al., 2019). We found that 980 of the 1007 genes had a human and at least one other species ortholog and a total of 1000 genes exhibited orthologs between at least two of the other 56 non-human species (Supplementary Table 2). We further calculated a conserved orthologs score (COS) for each gene based on the ortholog information in human and all other 56 non-human species (Supplementary Table 2). A comparison of COS and CES for 1007 genes indicates that highly conserved genes across the species (e.g., the DPT, LRRC4C, PCNA, and CTNNB1 genes) are more likely to have been recorded in fewer of the 15 data resources we used to generate the list of 1007 longevity and aging associated genes. However, the majority of the genes listed in more of the 15 resources (e.g., *APOE, TP53, WRN*, and *LMNA*) exhibit an intermediary ortholog score across 57 different species (Supplementary Figure 1). Since evolutionary-conserved genes exert a strong control on life-span and patterns of aging both in long and short-lived organisms (Finch and Tanzi, 1997), our results emphasize the importance of including genes with low CES scores to understand the genetic basis of longevity and aging in humans.

### Cross-Species Comparisons of Longevity and Aging-Related Genes Using PPINs

We explored the connections between the 1007 candidate longevity and aging associated genes using network and enrichment analysis methods, both within and across humans, mice, flies and worms. Network approaches have been shown promising for systems level understanding of longevity and the aging process in humans (Xue et al., 2007; Soltow et al., 2010; Levine and Crimmins, 2016). Network strategies that consider human protein–protein interactions (PPIs) can be used to highlight the potential importance of various pathways and hub genes in those networks, potentially linking human longevity and aging with and age-related diseases (Budovsky et al., 2007; Wolfson et al., 2009). In addition, human longevity and aging-related genes that are conserved across the PPIs of other non-human species that are known to confer increased longevity in those non-human species through loss of function mechanisms could be excellent targets for longevity and healthy-aging promoting interventions (Bell et al., 2009; Tacutu et al., 2012).

Initially, we retrieved all the physical PPIs for humans, mice, flies and worms based on the existing experimental evidences and constructed individual species-specific protein-protein interaction networks (PPINs) (Figure 1C). The number of proteins and interactions present in each species-specific PPIN are presented in Table 2. Next, we mapped the species-specific subsets of the 1007 longevity and aging–related genes and their corresponding orthologs to the respective species-specific PPINs. We identified 949 genes in human and 782, 309, and 200 human gene orthologs in mice, fly and worm, respectively, in the PPINs. We observed a substantial overlap of genes (*N* = 111) within the four species-specific PPINs (Figure 1D) which clearly indicates the existence of a conserved functionality of those genes associated across species.

**TABLE 2:** Physical protein-protein interaction network (PPIN) details for four species.

Species name	Number of proteins	Number of interactions
Human (*H. sapiens*)	17776	3300032
Mouse (*M. musculus*)	9825	36716
Fly (*D. melanogaster*)	9695	61392
Worm (*C. elegans*)	3770	11172

*The physical PPIs for human, mice, fly, and worm based on the existing experimental evidences from the Integrated Interactions Database (IID).*

**FIGURE 2:**
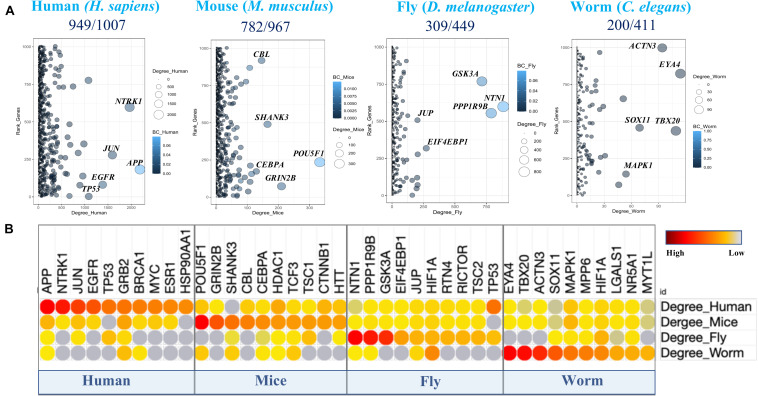
Cross-species PPIN topologies comparison. **(A)** The degree (connecting topology) distribution of aging and longevity related genes (Human) and their orthologs (mice, flies, and worms) in corresponding protein interactomes. The *Y* axis provides the ranking order based on the Convergent Evidence Score (CES) of the 1007 genes. The node size and color indicate the number of degree and betweenness centrality (BC) values respectively for each gene in the corresponding PPIN. **(B)** Top ten highly connected human aging and longevity related genes (hubs) in the protein interactome of each species and their corresponding orthologs in other species are highlighted.

The network connectivity (i.e., degree topology) of the genes within the PPINs was computed based on their first-order interacting partners in the individual PPINs. Further, “hub” genes in those networks were identified by comparing different network topology metrics associated with the genes (e.g., degree centrality and betweenness) together with the ranking of the genes based on their CES values in individual PPINs (Figure 2A). As expected, the human and mice PPIN topology showed higher connectivity among top ranked CES genes, whereas fly and *C. elegans* did not, likely due to poor orthology determination and complex evolutionary histories of the genes including gene gain or loss events, indicating that human and mouse genes have become involved in complex interactions and regulatory circuits (e.g., network “re-wiring”) over time. A comparison of the highly connected nodes among the top ten ranked proteins over the networks clearly indicates that “hub-like” proteins (e.g., APP, NTRK1, JUN, and EGFR) in humans potentially lose their hub-like properties in non-human species. However, certain proteins, like GRB2, HDAC1, H1F1A, and MAPK1, remain highly connected within the species-specific PPINs (Figure 2B). The existence of distinct conserved hub proteins in non-human species’ networks can be exploited for the identification of therapeutic targets in humans, so we therefore sought enriched biological pathways among the constructed PPINs for each species separately.

### PPIN-Specific Pathway Comparison Across the Species

Emerging evidence indicates that alterations in cellular signaling during the course of aging is important in controlling the lifespan in different species (Warner, 2005; Niedernhofer and Robbins, 2008). It has been also documented that the majority of the proteins involved in human longevity and aging are linked to signal transduction (Wolfson et al., 2009). Furthermore, the effects on lifespan of manipulations of orthologous lifespan-associated genes in different model organisms are, for the most part, similar across species, despite the evolutionary distances between them (Yanai et al., 2017; Möller et al., 2020).

**FIGURE 3:**
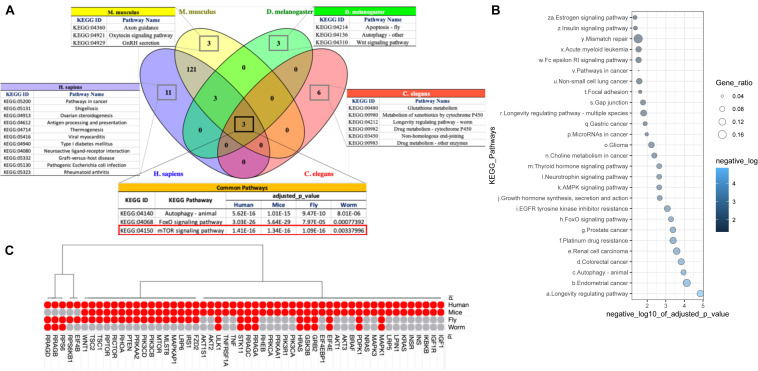
Cross-species pathways comparison. **(A)** Gene Set Enrichment Analysis (GSEA) of PPIN genes identified three KEGG pathways: Autophagy – animal (KEGG:04140), FoxO signaling pathway (KEGG:04068) and mTOR signaling pathway (KEGG:04150) are common within all four studied species. Pathways identified as species specific are also highlighted separately. **(B)** GSEA of common orthologs (111 genes) in PPIN of all four species identified 27 KEGG pathways with *P*-value threshold < 0.05. The size and color of each dot represent the adjusted *P*-value (after Bonferroni correction) and gene ratio, respectively for each pathway in the analysis. The gene ratio was calculated by subtracting the number of genes present in a particular pathway with the number of genes represent the respective pathway from our data. **(C)** The dot plot represents the number of genes from mTOR signaling pathway (KEGG:04150) for each species in our analysis. The present or absent of the orthologs within the species implies that the interaction of proteins involved in mTOR signaling pathway may be somewhat limited to each species.

We pursued functional and pathway enrichment analyses of the genes present in individual species-specific PPIN using Gene Ontology (GO) and Kyoto Encyclopedia of Genes and Genomes (KEGG) gene and pathway annotations. We found that three important KEGG signaling pathways, autophagy (KEGG:04140), FoxO signaling (KEGG:04068) and mTOR signaling (KEGG:04150), are common and highly enriched in all four species with an adjusted *p*-value ≤ 0.001 (Supplementary Table 4). Our results strongly indicate the involvement of genes within our list of 1007 longevity and aging-related genes in common signaling cascades across the species. We also observed some species-specific pathways that encompass genes in our list of 1007, such ovarian steroidogenesis, antigen processing and presentation and neuroactive ligand-receptor interaction in humans; axon guidance, oxytocin signaling pathway and GnRH secretion in mice; apoptosis and Wnt signaling pathway in fly; and glutathione metabolism, metabolism of xenobiotics by cytochrome P450 and Non-homologous end-joining in worms (Figure 3A). Such distinct pathways might be account for observed variability in aging in across the species. Our GO analysis also identified a total of 131 common biological processes across the four species that are significantly overrepresented. These highlight processes such as positive regulation of cell death, multicellular organism aging and determination of the adult lifespan – which are well-recognized molecular fingerprints of a longevity and aging (Supplementary Figure 2).

To probe whether the 3 conserved pathways (autophagy (KEGG:04140), FoxO signaling (KEGG:04068) and mTOR signaling (KEGG:04150), were due to the presence of shared genes within the species-specific PPINs, we performed an independent pathway enrichment analysis for the genes that were common in the PPINs of all the four species (*N* = 111) and did not find mTOR signaling among the 27 enriched KEGG pathways (Figure 3B and Supplementary Table 5). This implies that the interaction of proteins involved in mTOR signaling pathway may be somewhat specific to each species, or that a “rewiring” of certain networks has taken place during evolution. We therefore examined the interactions among genes for mTOR signaling pathways in each species (Figure 3C). A whole set of interactions presented by ribosome associated proteins, namely, EIF4B, RPS6KB1, RPS6 and regulators of cellular amino acid availability including RRAGB, RRAGD, were absent in, e.g., mice (Supplementary Figures 3A,B). In addition, the fruit fly’s mTOR signaling cascade lacked key interacting proteins in the human network related to glucose import and insulin signaling (Supplementary Figure 3C), while in *C. elegans*, only the interactions that involve regulation of cellular localization of macromolecules and response to starvation or nutrient depletion were identified (Supplementary Figure 3D). The evolutionary-conserved role of mTOR signaling network as longevity regulator is well documented. It is known that mTOR inhibition increases lifespan in model organisms, yet the precise mechanism through which this occurs is still poorly understood in humans (Weichhart, 2018). We observed a notable alteration in a number of network proteins in mTOR signaling pathways across the 4 different species. Our study suggests an evolutionary-mediated progressive gain of function for certain genes in mTOR signaling pathways which leads to increased functional complexity, and therefore could increase vulnerability to death and thus limit lifespan in humans.

### “Local-PPIN” Analysis: Exploring Sub-Networks Within PPIs

**FIGURE 4:**
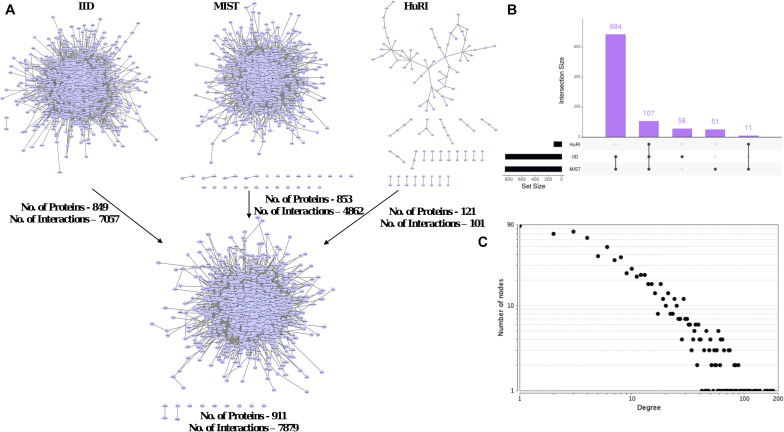
Local-PPIN of aging and longevity related genes in human. **(A)** Three human protein interactomes – IID (Kotlyar et al., 2019), MIST (Hu et al., 2018), and HuRI (Luck et al., 2020) were compared to map the interactions within the aging and longevity related genes. A union of all above three interactomes identified 911 genes are physically interacted with each other at protein level (7879 local-interactions). **(B)** Overlapping of aging and longevity related genes in three human interactomes present in the local-PPIN. **(C)** A scale-free degree distribution graph for 911 genes in the local-PPIN related to aging and longevity.

We explored “local-PPINs” that represent sub-networks within the human protein interactome and whether they were highly enriched with genes among the list 1007 longevity and aging- related genes (Figure 4A). The physical PPI information from three major human interactomes (IID, MIST, and HuRI) was gathered and combined together in order to overcome the incompleteness in the characterization of human protein interactome associated with any one of the three (Kovács et al., 2019). We found a total of 911 proteins and 7879 known interactions are present in local-PPINs where the majority of those proteins (107 within all three, 684 between IID and MIST) are consistent across the databases (Figure 4B), which increased our confidence in the use of our constructed network for further analysis.

Our assessment of the overall network topology revealed a scale-free degree distribution among the nodes in the local-PPIN (Figure 4C). Such a topological configuration enables a highly robust network against random perturbations (Barabási and Albert, 1999). We also observed that genes related to longevity and aging are not randomly distributed in the human interactome, but rather exhibit a precise interconnectivity regardless the size of their local-PPI network. It has been shown that proteins associated disease with similar symptoms or origins, as well as related phenotypes in general, have an increased tendency to interact with each other (Barabási et al., 2011). We find potential analogous relationships among longevity and aging-related genes in our study, which indicates a functionally conserved role of the local-PPIN in human longevity and aging. We found that hub-like properties of a few proteins like TP53, EGFR, APP, NTRK, and JUN remain un-changed in the local-PPIN when considered with the entire human interactome (Figure 2B). To corroborate these findings, we compared the topological parameter of the 1007 studied genes in all three human interactomes (IID, MIST, and HuRI) along with the local-PPIN (Supplementary Figure 4A). We observed that different topological properties (like degree and betweenness) of the hub-like genes remain preserved in the local-PPIN (Supplementary Figures 4B,C). Based on our analyses, it appears that the hub-like genes maintain contact either directly or indirectly with most of the nodes in a network – the implication being that the removal of such hubs could significantly impact the overall network topology or even lead to a lethal phenotype as shown by others (Jeong et al., 2001; Ferrarini et al., 2005). The majority of these hub-like genes are widely recognized for their importance in cancer and other age-related diseases like Alzheimer’s disease (Ershler and Longo, 1997; Lazarov et al., 2010; Rolyan et al., 2011; Aunan et al., 2017). Genes like *EGFR*, *APP*, and *NTRK* encode cell surface receptor proteins and often serve as key switches between different signaling pathways and sustain a cross-talk between the pathways and their coordinated functions. Also, *TP53* and *JUN* encode active transcription factors and regulate the expression for a diverse array of genes in humans. Topologically rich properties of the hub-like genes in the local-PPIN suggest a regulatory role of those genes in the longevity and aging mediated pathways and biological processes in humans. Altogether, our study highlighted that the hub-like proteins could offer the potential of being the primary targets for longevity-promoting interventions.

### Comparison of Human Tissue Specific Local-PPIN for the Aging and Longevity Related Genes

We further examined the abundance of the local-PPIN in different human tissues, which provides an obvious vehicle for studying conserved gene expression patterns since all human tissues originate from a single fertilized cell during embryogenesis and yet differ immensely in function (Lukk et al., 2010). Tissue-specific transcript expression profiling highlighted an elevated mRNA level for many of our putative longevity and aging genes in human liver, heart, and skeletal muscle (Supplementary Figure 5). The consistency of this finding in from GTEx and HPA database clearly indicates a potential tissue-specific role of those genes human longevity and aging.

**FIGURE 5:**
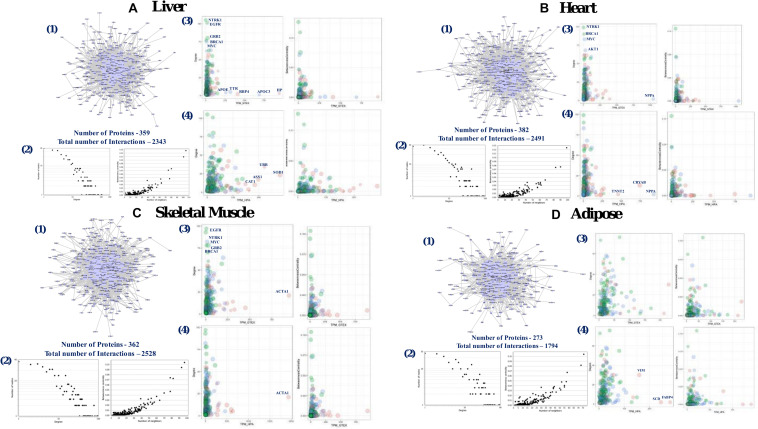
Tissue specific local-PPIN of human aging and longevity related genes. Tissue specific local-PPIN was constructed based on protein level expression information for aging and longevity related genes in human: **(A)** liver, **(B)** heart, **(C)** skeletal muscle, and **(D)** adipose tissues. [1] The network graphs indicate the number of proteins expressed in each tissue and their interactions. [2] The degree and betweenness centrality distribution plots for tissue specific local-PPINs. [3] Comparison of transcripts and protein level expression values of genes present in tissue specific local-PPIN based on Genotype-Tissue Expression (GTEx) data. [4] Comparison of transcripts and protein level expression values of genes present in tissue specific self-PPIN based on The Human Protein Atlas (HPA) data. The node size represents the Convergent Orthologs Score (COS) of genes calculated based on cross-species orthologs statistics in 57 different species from OrthoDB database. The node color represents the protein level expression values of genes in respective tissues.

**FIGURE 6:**
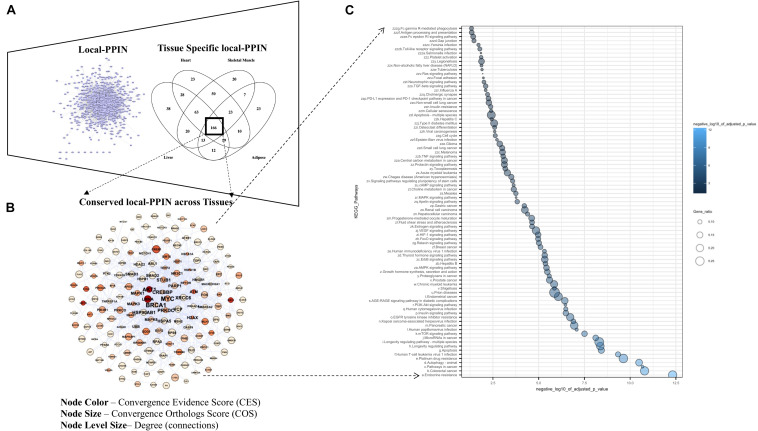
Inter-tissue pathways associated with conserved module of local-PPIN. **(A)** Comparisons of tissue specific local-PPINs confirmed an overlapping of 166 aging and longevity related genes across four different human tissues (liver, heart, skeletal muscle, and adipose). **(B)** A total of 701 defined local-PPIs between 160 genes are conserved across all four studied local-PPIN. The node color and size represent the Convergent Evidence Score (CES) and Convergent Orthologs Score (COS) of genes respectively. The node level size indicates the connectivity statistics (degree value) of genes in the conserved local-PPIN across different human tissues. **(C)** The KEGG pathway enrichments for 160 genes in conserved local-PPIN across tissues highlighted 160 different pathways with *P*-value threshold < 0.05. The *X*-axis represents the negative log10 of the adjusted *P*-values (after Bonferroni correction) for each pathways term. The node size is corresponding to the Gene ratio which was calculated by subtracting the number of genes present in a particular pathway with the number of genes represent the respective pathway from our data.

We developed four different tissue-specific local-PPINs constructed from the protein level expression information of genes from human liver, heart, skeletal muscle, and adipose tissues (Figure 5). We found comparatively low tissue specificity for majority of the 1007 genes in adipose tissue at the mRNA expression level, and thus included an adipose tissue specific local-PPIN in our analysis to observe the functional variability of out longevity and aging-related genes in tissue-specific physical PPINs. All four tissue-specific local-PPINs essentially represent sub-networks of the human interactome and we find that our 1007 genes are predominantly overrepresented in these PPINs. We noticed that nearly one-third of the proteins, i.e., 399, 382, 362, and 273 from the local-PPIN, are present in the four tissue-specific networks of liver, heart, skeletal muscle and adipose, respectively. We also located a conserved module of PPIs among the aging and longevity-related genes based upon their protein expression in various tissues (Figure 6A).

Given that the mRNA levels and protein levels of a gene do not generally correlate due to post transcriptional and post-translational modifications, we correlated the mRNA transcript levels of the longevity and aging genes to their actual translated protein levels across four different human tissues (Supplementary Figure 6). We observed that genes with moderate expression at both the transcript and protein level are highly correlated in tissues whereas a relatively low correlation was observed as compared to both the high- and low-level genes in the tissue-specific PPINs. In addition, a comparison of different topological features of the networks highlighted a consistently “scale-free” topology for all the four tissue-specific networks in our study (Figures 5A–D). We noticed that the highly expressed proteins did not tend to be hub proteins in tissue-specific networks. We found that highly tissue-specific genes in liver (*SOD1*, *HP*), heart (*NPPA*, *CRYAB*), skeletal muscle (*ACTA1*) and adipose (*FABP4*, *SCD*, and *VIM*) are not hubs in the respective tissue networks (Figures 5A–D). Studies have also suggested that the tissue-specific genes are not highly connected in their corresponding tissue network, but the findings are not necessarily conclusive for various reasons (Pierson et al., 2015; Sonawane et al., 2017). Altogether, our study pinpointed a number of non-tissue-specific hubs in tissue-specific networks that are relevant for local-PPIN dependent functionality in human longevity and aging.

### Inter-Tissue Pathways Associated With Conserved Modules Within the Local-PPIN

In addition, we identified an overlapping module of 701 defined PPIs implicated 160 genes which remain conserved across all four tissue-specific local-PPINs in our study (Figure 6B). A number of genes, including *AKT1*, *BRCA1*, *MYC*, and *CREBP*, show an enriched connectivity in the inter-tissue local-PPIN based upon their protein level expression profiling in various tissues. Accumulating the gene level evidence (CES) and orthologs (COG) information together with the network properties, we have highlighted a couple of genes, such as *AKT1*, *LMNA*, and *MYC*, as important candidates in the conserved local-PPIN for different tissue-specific networks likely implicated in human longevity and aging.

We explored the functionality of those 160 conserved genes in the inter-tissue local-PPIN in a pathway enrichment analysis and identified a total of 85 different KEGG pathways that are enriched above the defined threshold cut-off (Figure 6C). We noticed that nearly all the pathways that were identified in common inter-species PPIN (Supplementary Table 5) and human inter-tissue local-PPIN (Supplementary Table 6) overlap, revealing FoxO signaling and autophagy as shared biological processes that seem to be conserved across different species and human tissue within this PPIN. A significant enrichment of mTOR signaling pathway in the local-PPIN across different tissues clearly indicates a distinct connectivity for a group of genes associated with the key process of mTOR signaling in human. The mTOR pathway is crucial for nutrient sensing, and its importance in longevity is well-known. Partial suppression of the mTOR axis across a variety of organisms has been seen to prolong the lifespan and delay the onset of age-related diseases (Weichhart, 2018). Integrated analysis based on cross-species and cross-tissue PPINs such as this have been shown to reveal longevity and aging related biological process and identify key longevity-enhancing drug targets in humans (Avelar et al., 2020). Together our findings illuminate remarkable inter-species and human inter-tissue differences in the protein interactions involved in mTOR signaling that can serve as primary targets for enhancing longevity in humans.

### Cross-Species Longevity Drug Targets Comparison

**FIGURE 7:**
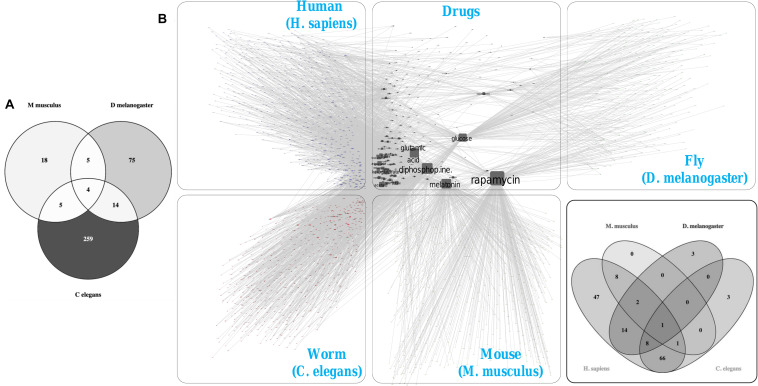
Cross-species longevity drug targets mapping. **(A)** Compilation the drugs, compounds and supplements (including natural products and nutraceuticals) with anti-aging properties that extend longevity in three model organisms (mice, flies, and worms) from DrugAge database. **(B)** The species-specific drug targets mapping for 380 identified drugs in human, mice, flies and worms using available drug-protein interaction information from STITCH database. The Venn diagram shows the overlapping of drugs based on shared targets within all four studied species.

In order to test the translational potential of our findings, we created a list of reported drugs and compounds which have been experimentally verified to promote longevity in the non-human species we considered (i.e., mice, fruit fly and *C. elegans*) (Figure 7A). We found there are a total four compounds (Metformin, N-acetyl-L-cysteine, Rapamycin and Vitamin E) which are commonly reported as longevity-enhancing molecules in all three species (Supplementary Table 7).

We identified the potential targets of the aforesaid drugs for each species, including humans, using available drug-protein interaction information (Figure 7B). Using experimental evidence for protein–chemical interactions, we identified the top protein targets for each species and mapped them to the respective species-specific PPINs (Supplementary Figure 7). Certain hub proteins (APP and EGFR) in the human PPIN were observed to be the putative targets for multiple anti-aging drugs. The target proteins of the FDA approved drug rapamycin were found to be conserved across all four species (Figure 7B). Rapamycin is a specific inhibitor of mTOR and has been shown to prolong the lifespan in different organisms; however, its use is somewhat problematic in humans due to underlying side-effects (Papadopoli et al., 2019). Drugs like melatonin (DB01065) and metformin (DB00331) were determined to share common targets with rapamycin in the human PPIN we constructed, including MTOR, IRS1, IL2, and AKT1 (Supplementary Figure 8). A recent study has shown the inhibitory mechanism of metformin in mTOR-mediated cellular control, and metformin is also the first drug approved by the FDA to enter a clinical trial to assess its effect on prolongation of a healthy lifespan in humans (Amin et al., 2019). Together our results pinpointed a couple of potential drugs and targets in the protein–chemical interactions network which could be considered to design an effective combination therapy for longevity enhancement in humans.

### Data Validation

**FIGURE 8:**
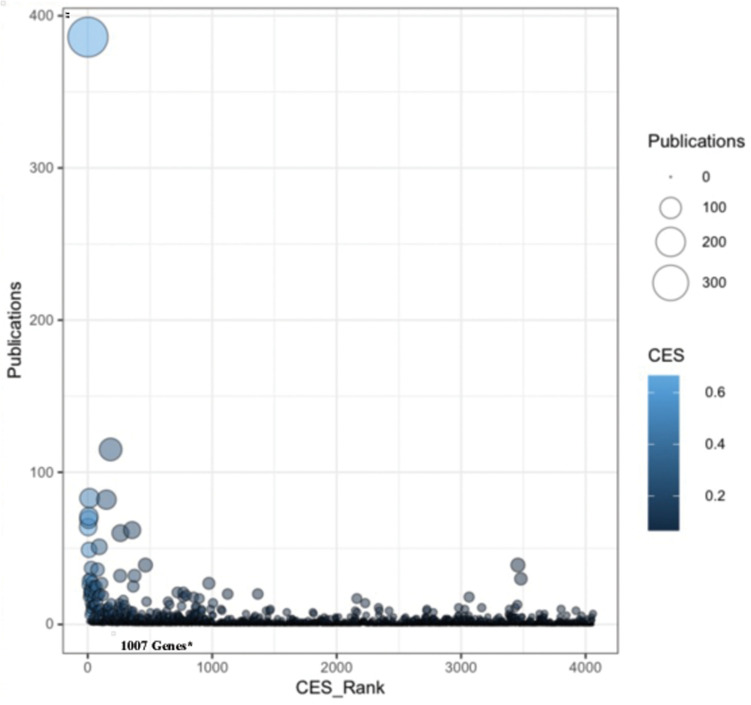
Literature-based mapping of aging and longevity related genes. A gene to literature-based mapping identified the available PubMed literatures evidences for the studied genes to verify their links with human longevity and aging. The *X*-axis represents the Convergent Evidence Score (CES) based rank (node color) and the *Y*-axis represents the number of PubMed literature counts (node size) for each gene in our study.

We manually curated fifteen key data resources related to aging and longevity and identified 4060 non-redundant genes. Further, we selected 1007 genes as potential candidates through data harmonization efforts and performed a number of complementary integrated analyses. To verify the selected longevity and aging-related gene-list we used in our study, we performed a gene to literature-based mapping and identified the significant contribution of each gene related to human longevity and aging. A total of 1483 genes were mapped with the available PubMed literature to verify their links with human aging and longevity (Figure 8). We found that majority of the genes in the selected gene-list (1007 genes) are key contributors in terms of PubMed literature counts for human longevity and aging (Supplementary Table 8). Genes such as *APOE*, *APP*, *SIRT1*, *IGF1*, and *IL6* have the highest number of literature citations and are also present on the top of our selected gene-list and also exhibit high CES values, which validates our CES ranking to some degree. On the other hand, we did not include some genes, such as *CRP* (CES Rank: 3456), *COMT* (CES Rank: 3480), *VDR* (CES Rank: 1124), because they had low CES ranking, yet are potential candidate genes for longevity and aging based on the literature. Obviously, studies that consider different criteria and curation strategies for identifying genes associated with longevity and aging are needed, as well as studies that accommodate new genes found to be associated with longevity and aging.

## Conclusion

We considered a study of the connections between genes shown to be associated with longevity and aging. These connections included those arising from their interactions in protein-protein interaction networks (PPINs), expression patterns across different tissues, as well as their evolutionary-mediated connections as orthologs across species. Our findings shed light on the remarkable inter-species and human inter-tissue differences in the protein interactions, particularly involved in mTOR signaling, a key process for nutrient sensing known to mediate longevity and aging. Ultimately, our work provides a paradigm for how researchers can study genes related to human lifespan and aging using systems biology and evolutionary analyses and could lead to potential targets for longevity-enhancing drugs.

## Data Availability Statement

The original contributions presented in the study are included in the article/Supplementary Material, further inquiries can be directed to the corresponding author.

## Author Contributions

AP and NS conceived the study. AP performed the data curation and analysis. AR contributed to shiny app development. AP and AR wrote the manuscript. NS participated in revising it critically for important intellectual content and gave final approval of the version to be submitted. All authors contributed to the article and approved the submitted version.

## Conflict of Interest

The authors declare that the research was conducted in the absence of any commercial or financial relationships that could be construed as a potential conflict of interest.

## Publisher’s Note

All claims expressed in this article are solely those of the authors and do not necessarily represent those of their affiliated organizations, or those of the publisher, the editors and the reviewers. Any product that may be evaluated in this article, or claim that may be made by its manufacturer, is not guaranteed or endorsed by the publisher.

## Footnotes

1
https://biit.cs.ut.ee/gprofiler

2
https://agingnetwork.shinyapps.io/frontiers/

## Supplementary Material

Supplementary Figure 1 Comparison between CES and COS for 1007 aging and longevity related genes. The *X*-axis represents the Convergent Evidence Score (CES) and *Y*-axis represents the Convergent Orthologs Score (COS) for all 1007 human aging and longevity related genes included in the study. The node color and size correspond to the number of available database evidences and number of species available with the orthologs of each gene, respectively.

Supplementary Figure 2 Cross-species Gene Ontology (GO) comparison. Gene Set Enrichment Analysis (GSEA) of PPIN genes identified 131 GO biological process (GO-BP) are common within all four studied species with *P*-value threshold < 0.05. The size and color of each dot represent the adjusted *P*-value (after Bonferroni correction) and gene ratio, respectively for each GO-BP term in the analysis. The gene ratio was calculated by subtracting the number of genes present in a particular GO-BP with the number of genes represent the respective term from our data.

Supplementary Figure 3 Cross-species - mTOR signaling pathway (KEGG:04150) comparison. The species-specific KEGG mTOR signaling pathway (KEGG:04150) for **(A)** human [Hsa04150], **(B)** mice [Mmu04150], **(C)** flies [Dme04150], and **(D)** worms [Cel04150]. The human aging and longevity related genes and their orthologs were mapped in each species (highlighted - red).

Supplementary Figure 4 Global vs local-PPIN properties comparison. **(A)** Comparison of topological degree of 1007 studied genes in all three human interactomes (IID, MIST and HuRI) along with the local-PPIN. The topological distributions - **(B)** Degree and **(C)** betweenness Centrality (BC) of the highly connected nodes (top 20) indicate a global hub-like properties of many human aging and longevity related genes present in the local-PPIN.

Supplementary Figure 5 Tissue-specific transcripts distributions. The Transcripts Per Million (TPM) distributions were plotted to identify the transcript level abundance of aging and longevity related genes in multiple human tissues from **(A)** Genotype-Tissue Expression (GTEx) and **(B)** The Human Protein Atlas (HPA) database. We identified the normalized expression values (TPM-NX) for 987 and 1001 genes in 34 and 43 different human tissues, respectively from both the database. Elevated transcript level expressions were observed in heart, skeletal muscle and liver tissue in both the datasets.

Supplementary Figure 6 Comparison of the transcript and protein expression profiling. Both the transcript and protein level expression of genes were compared in four different human tissues: **(A)** liver, **(B)** heart, **(C)** skeletal muscle, and **(D)** adipose using the data from Human Protein Atlas (HPA) database. The *X*-axis indicates the normalized TPM values for the transcripts abundant and the *Y*-axis indicates the protein level expression values – Strong (1), moderate (0.50), weak (0.25), and negative (0) or no expression. To obtain the tissue expression of the proteins, we used the primary cell type specific expression (staining intensity) of the proteins.

Supplementary Figure 7 Cross-species PPIN topologies comparison for longevity drug targets. The degree (connecting topology) distribution of aging and longevity related genes (human) and orthologs (mice, flies, worms) in corresponding protein interactomes. The *Y* axis provides the ranking order based on the Convergent Evidence Score (CES) of the 1007 genes. The node size indicates the number of degree associated with a gene in the corresponding PPIN. The node color represents the designated status of the gene as drug target (1 – mapped and 0 – No mapped) for the longevity associated drugs.

Supplementary Figure 8 Potential candidates in cross-species longevity drug-targets interaction network. Species-specific targets for the drugs like rapamycin, melatonin, and metformin were represent through an interactive drug-target interaction network. The color of the nodes represents different species like purple (human), yellow (mice), green (flies) and red (worm). The shape of the nodes indicates – targets (round) and drugs (square). Share common targets of those drugs in human PPIN were highlighted, including MTOR, IRS1, IL2, and AKT1.
