# Supplementary figures and images for: Cross-Species and Human Inter-Tissue Network Analysis of Genes Implicated in Longevity and Aging Reveal Strong Support for Nutrient Sensing
Source: Front Genet. 2021 Aug 27;12:719713. doi: 10.3389/fgene.2021.719713 (PMC8430347; doi:10.3389/fgene.2021.719713)

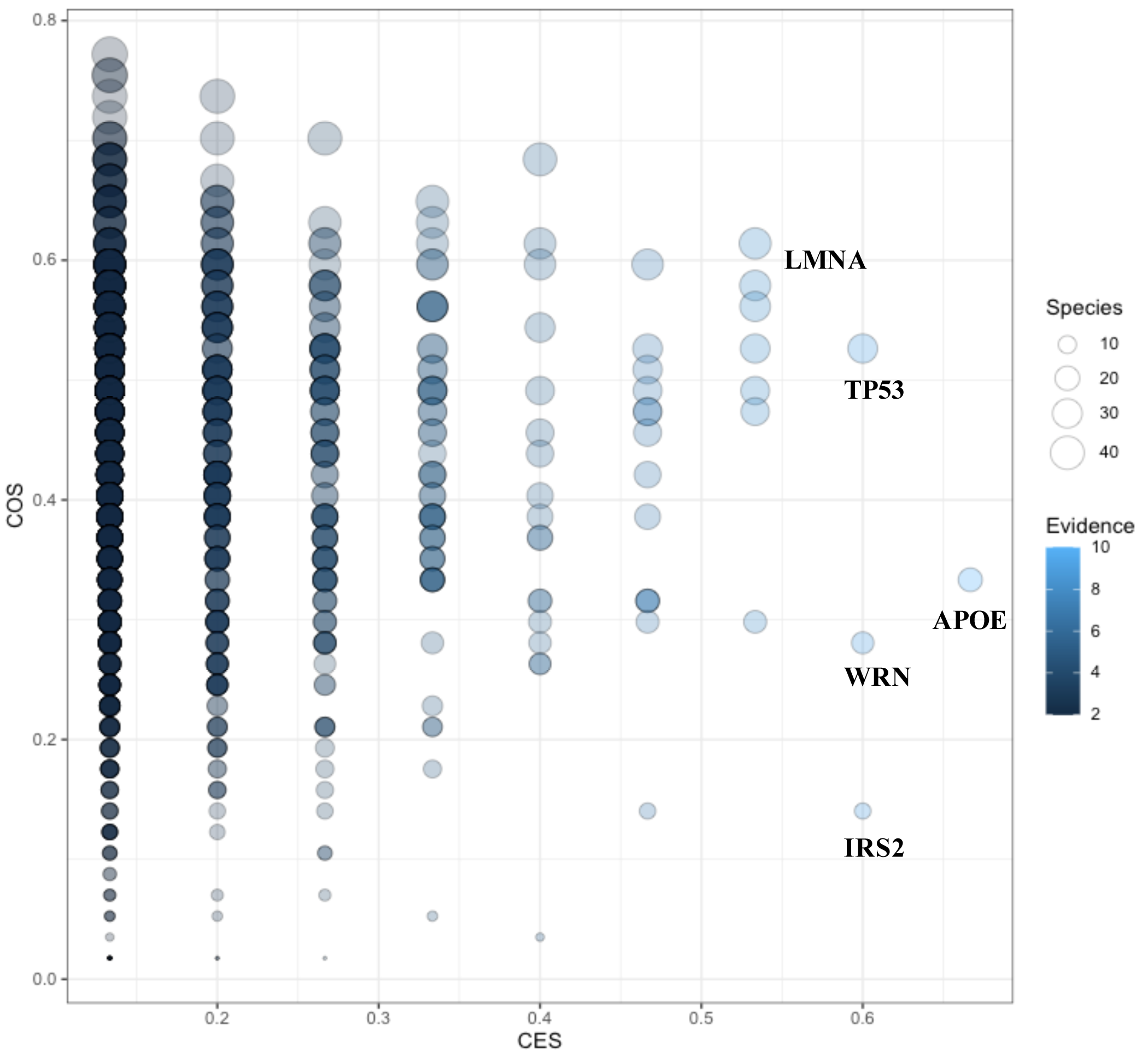

Supplement: Supplementary Figure 1 — Comparison between CES and COS for 1007 aging and longevity related genes. The X-axis represents the Convergent Evidence Score (CES) and Y-axis represents the Convergent Orthologs Score (COS) for all 1007 human aging and longevity related genes included in the study. The node color and size correspond to the number of available database evidences and number of species available with the orthologs of each gene, respectively. [file Image_1.TIF]

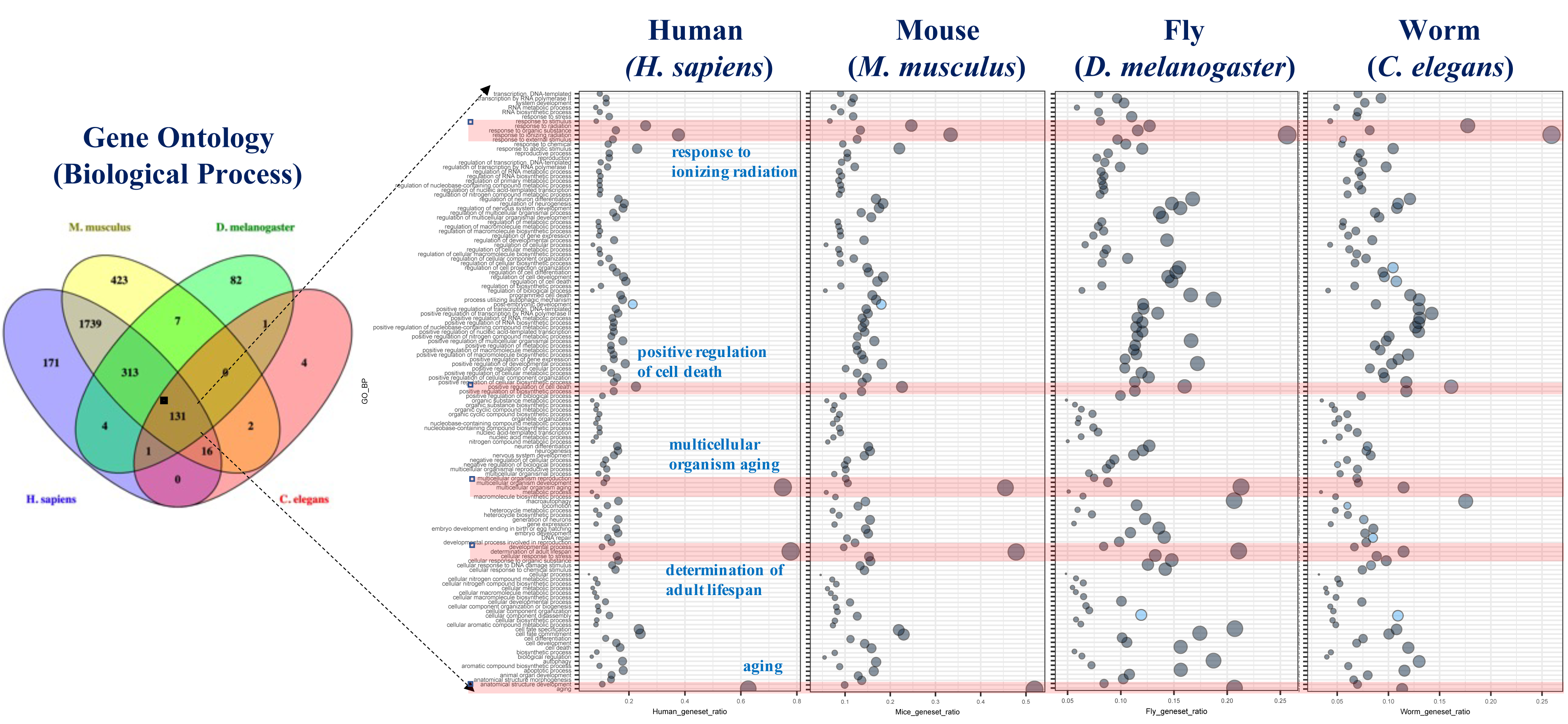

Supplement: Supplementary Figure 2 — Cross-species Gene Ontology (GO) comparison. Gene Set Enrichment Analysis (GSEA) of PPIN genes identified 131 GO biological process (GO-BP) are common within all four studied species with P-value threshold < 0.05. The size and color of each dot represent the adjusted P-value (after Bonferroni correction) and gene ratio, respectively for each GO-BP term in the analysis. The gene ratio was calculated by subtracting the number of genes present in a particular GO-BP with the number of genes represent the respective term from our data. [file Image_2.TIF]

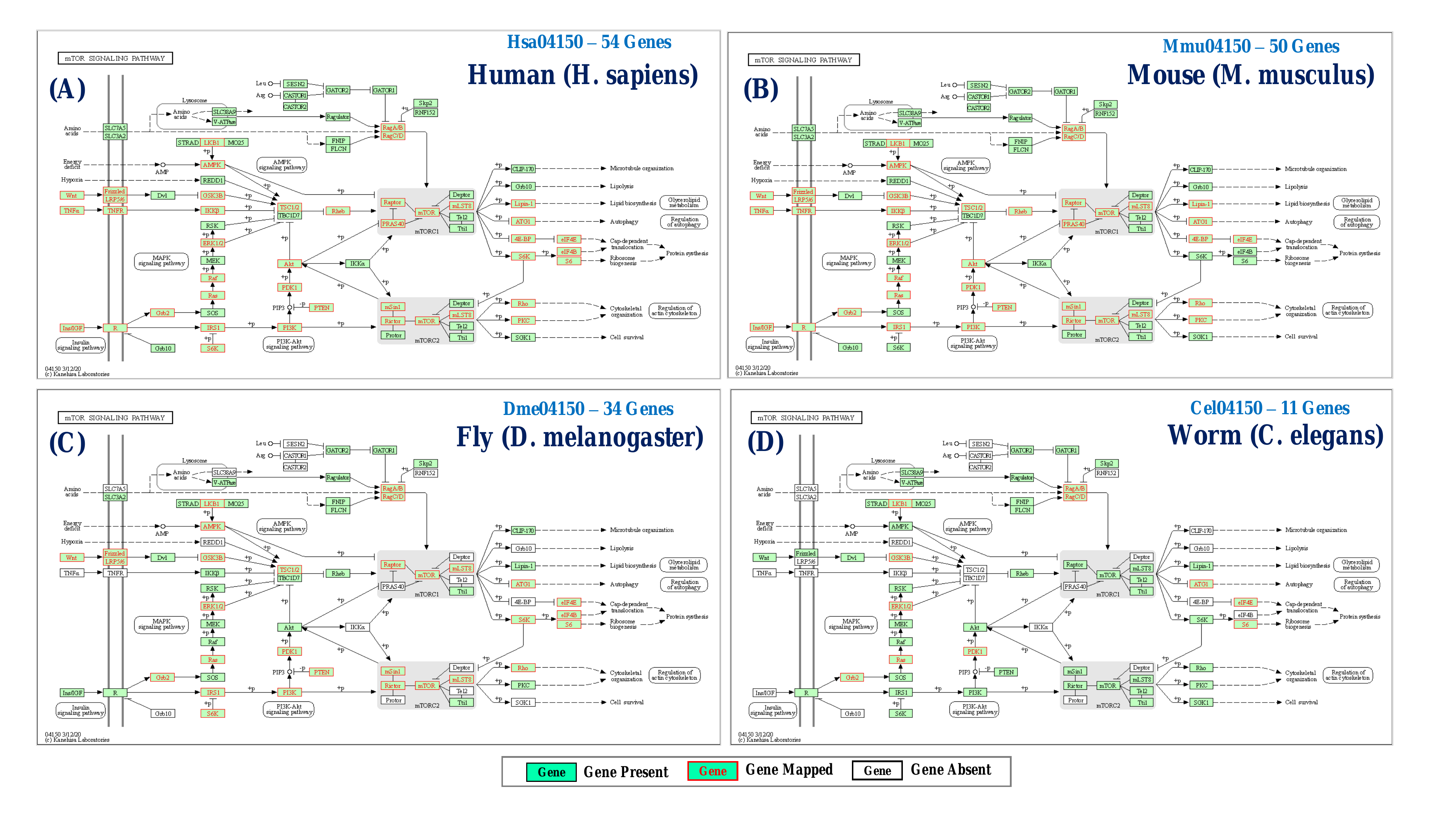

Supplement: Supplementary Figure 3 — Cross-species - mTOR signaling pathway (KEGG:04150) comparison. The species-specific KEGG mTOR signaling pathway (KEGG:04150) for (A) human [Hsa04150], (B) mice [Mmu04150], (C) flies [Dme04150], and (D) worms [Cel04150]. The human aging and longevity related genes and their orthologs were mapped in each species (highlighted - red). [file Image_3.TIFF]

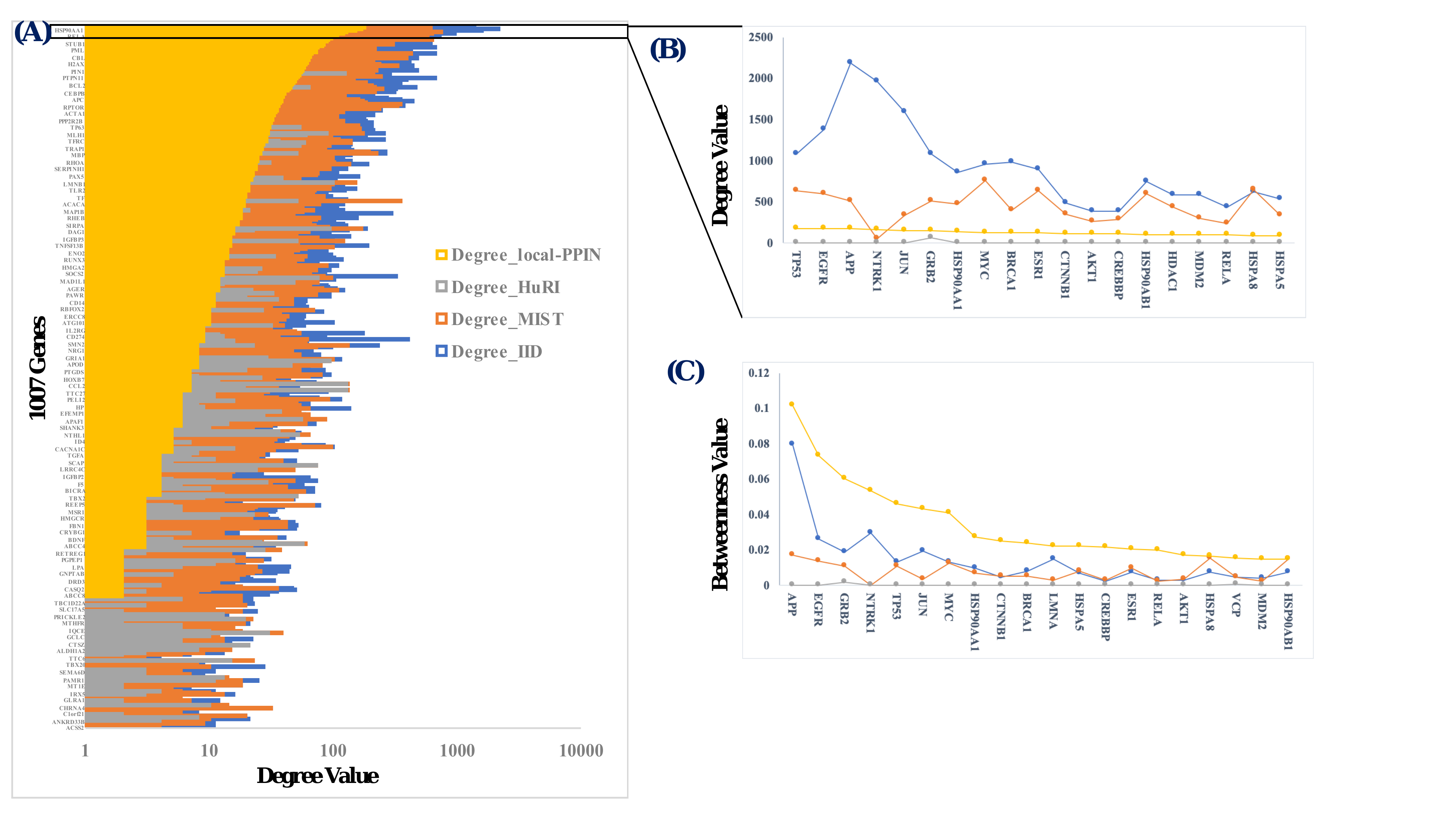

Supplement: Supplementary Figure 4 — Global vs local-PPIN properties comparison. (A) Comparison of topological degree of 1007 studied genes in all three human interactomes (IID, MIST and HuRI) along with the local-PPIN. The topological distributions - (B) Degree and (C) betweenness Centrality (BC) of the highly connected nodes (top 20) indicate a global hub-like properties of many human aging and longevity related genes present in the local-PPIN. [file Image_4.TIFF]

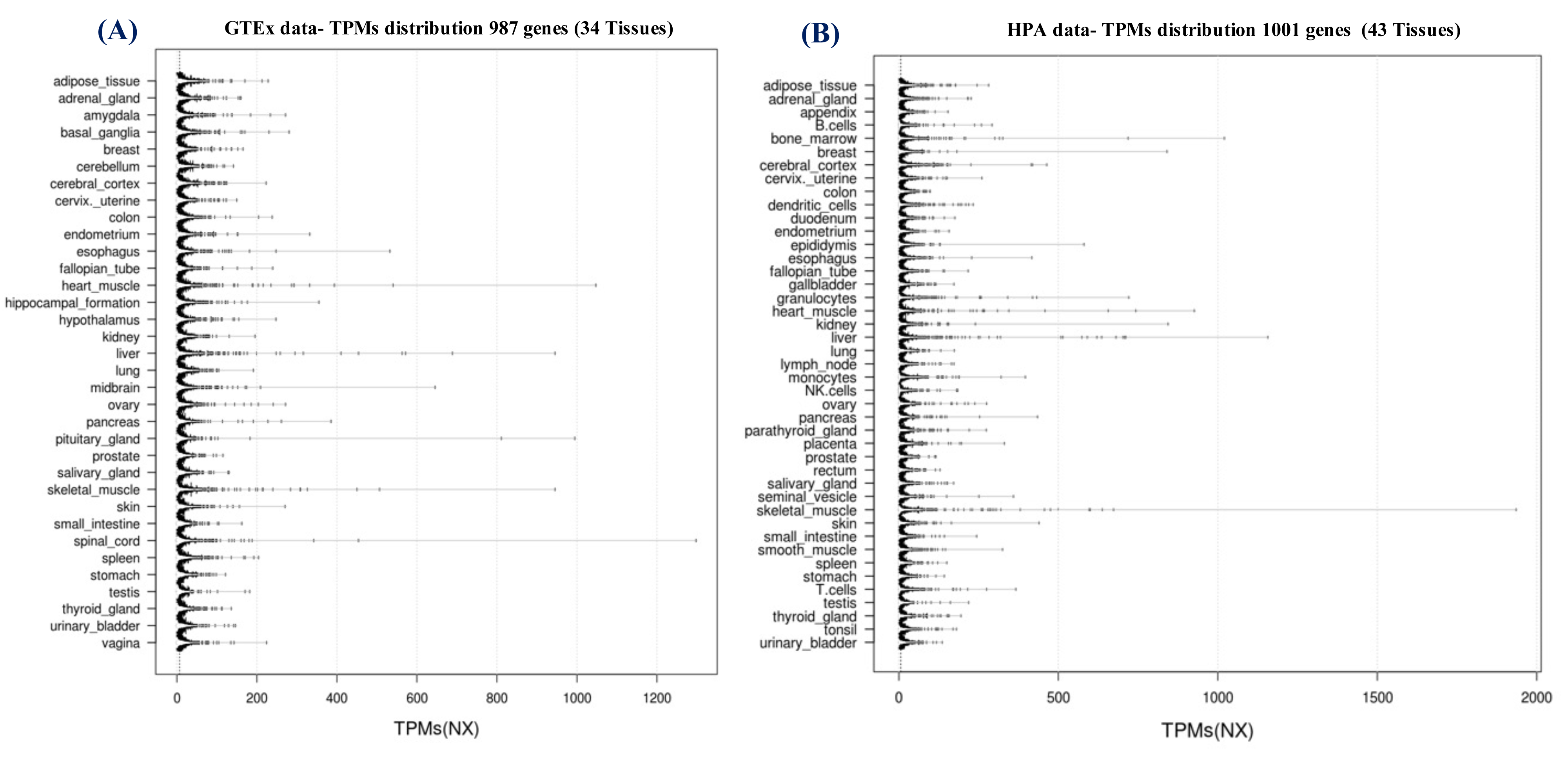

Supplement: Supplementary Figure 5 — Tissue-specific transcripts distributions. The Transcripts Per Million (TPM) distributions were plotted to identify the transcript level abundance of aging and longevity related genes in multiple human tissues from (A) Genotype-Tissue Expression (GTEx) and (B) The Human Protein Atlas (HPA) database. We identified the normalized expression values (TPM-NX) for 987 and 1001 genes in 34 and 43 different human tissues, respectively from both the database. Elevated transcript level expressions were observed in heart, skeletal muscle and liver tissue in both the datasets. [file Image_5.TIF]

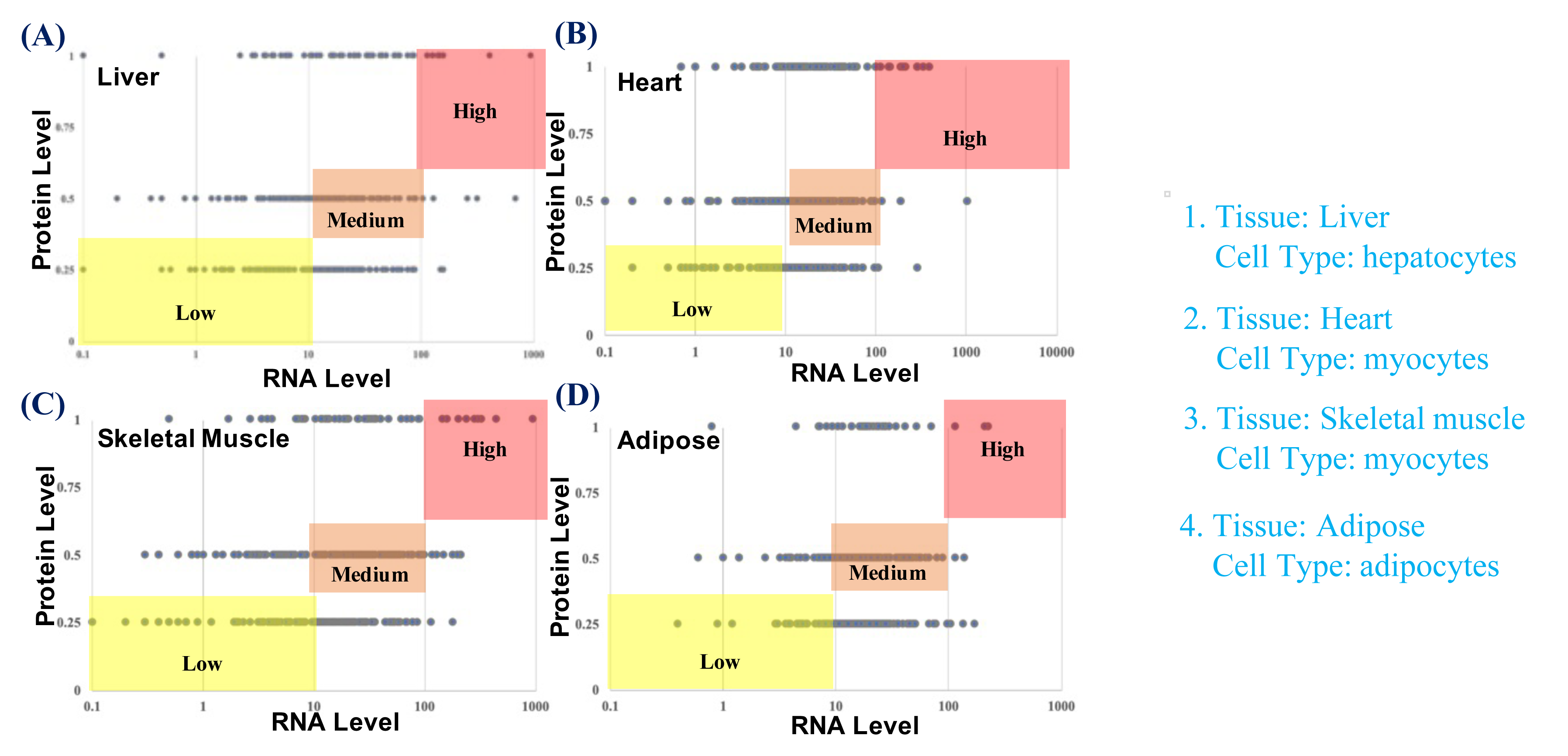

Supplement: Supplementary Figure 6 — Comparison of the transcript and protein expression profiling. Both the transcript and protein level expression of genes were compared in four different human tissues: (A) liver, (B) heart, (C) skeletal muscle, and (D) adipose using the data from Human Protein Atlas (HPA) database. The X-axis indicates the normalized TPM values for the transcripts abundant and the Y-axis indicates the protein level expression values – Strong (1), moderate (0.50), weak (0.25), and negative (0) or no expression. To obtain the tissue expression of the proteins, we used the primary cell type specific expression (staining intensity) of the proteins. [file Image_6.TIF]

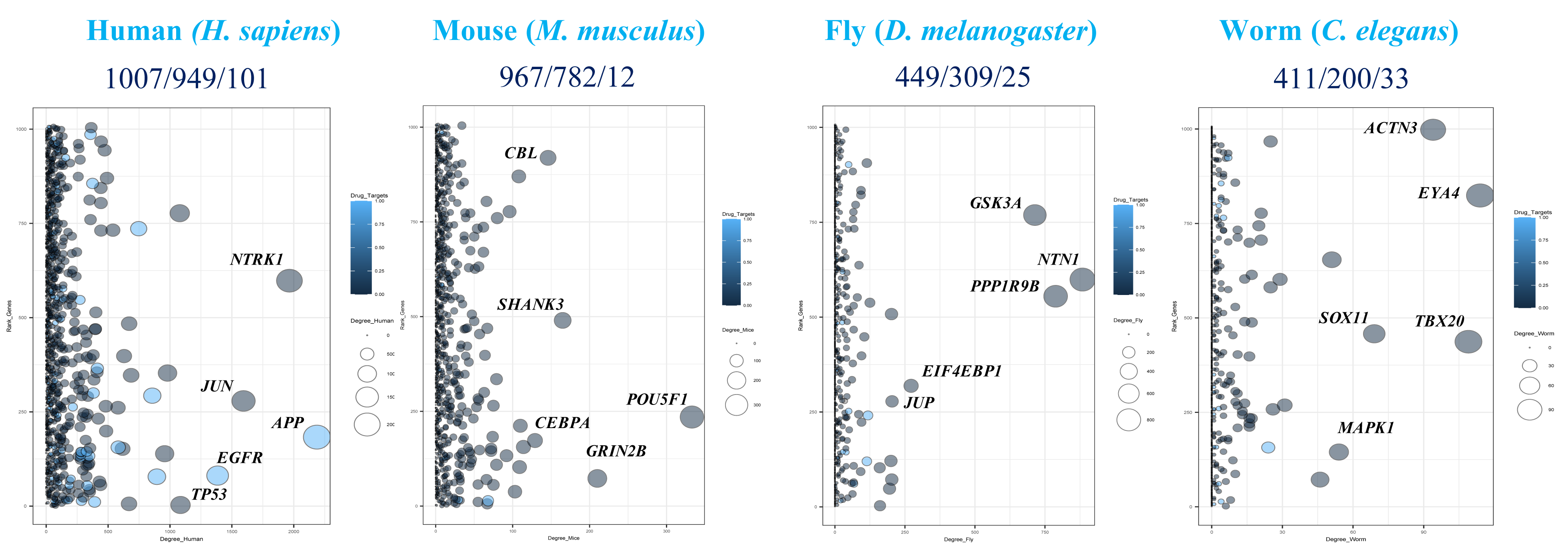

Supplement: Supplementary Figure 7 — Cross-species PPIN topologies comparison for longevity drug targets. The degree (connecting topology) distribution of aging and longevity related genes (human) and orthologs (mice, flies, worms) in corresponding protein interactomes. The Y axis provides the ranking order based on the Convergent Evidence Score (CES) of the 1007 genes. The node size indicates the number of degree associated with a gene in the corresponding PPIN. The node color represents the designated status of the gene as drug target (1 – mapped and 0 – No mapped) for the longevity associated drugs. [file Image_7.TIF]

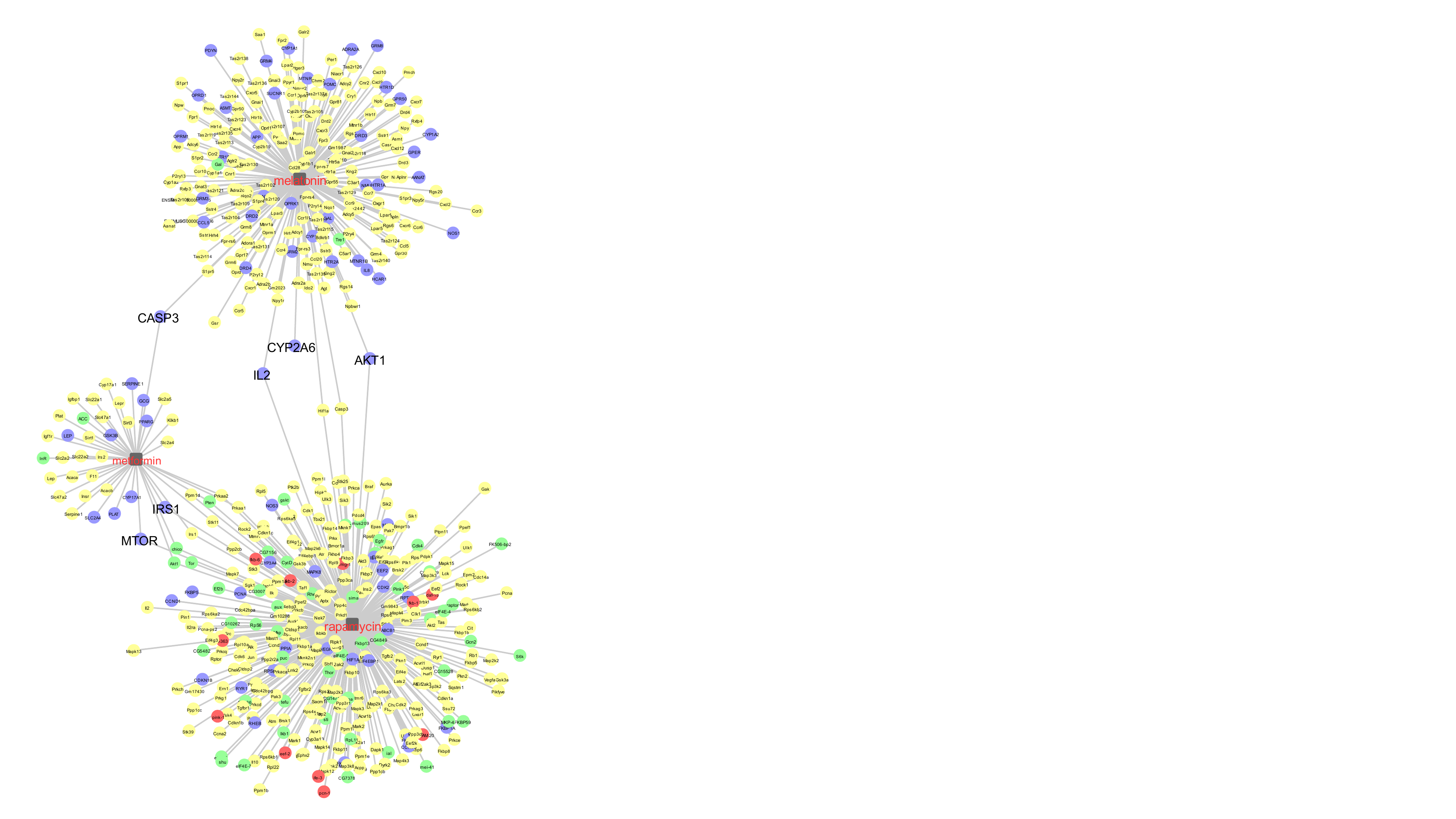

Supplement: Supplementary Figure 8 — Potential candidates in cross-species longevity drug-targets interaction network. Species-specific targets for the drugs like rapamycin, melatonin, and metformin were represent through an interactive drug-target interaction network. The color of the nodes represents different species like purple (human), yellow (mice), green (flies) and red (worm). The shape of the nodes indicates – targets (round) and drugs (square). Share common targets of those drugs in human PPIN were highlighted, including MTOR, IRS1, IL2, and AKT1. [file Image_8.TIFF]
